# The Gradient Heterogeneity of Deserts Alters the Interaction Relationships Between Xerophytic Plants and Soils

**DOI:** 10.3390/biology14081048

**Published:** 2025-08-14

**Authors:** Jinlong Wang, Yudong Chen, Xiaotong Li, Xiaojuan Cao, Hongli Tang, Guanghui Lv

**Affiliations:** 1 College of Ecology and Environment, Xinjiang University, Urumqi 830017, China; ydaccepted@sina.com (Y.C.); lixiaotong@stu.xju.edu.cn (X.L.); cxj019085@163.com (X.C.); 17686206367@163.com (H.T.); ler@xju.edu.cn (G.L.); 2 Key Laboratory of Oasis Ecology of Education Ministry, Xinjiang University, Urumqi 830017, China; 3 Xinjiang Jinghe Observation and Research Station of Temperate Desert Ecosystem, Ministry of Education, Jinghe 833300, China

**Keywords:** desert ecosystem, rhizosphere, functional traits, microbial community structure, co-occurrence network

## Abstract

Desert ecosystems pose extreme challenges to plant survival. This study explores the adaptive strategies of two xerophytic halophytes, *Alhagi sparsifolia* and *Nitraria roborowskii*, in Xinjiang’s Ebinur Lake wetland, focusing on their plant–soil–microbe (PSM) coupling systems across desert gradients. Results revealed significant interspecific and gradient-dependent differences in plant functional traits: *A. sparsifolia* showed high growth plasticity with a fast-growth strategy, while *N. roborowskii* adopted a conservative strategy. Rhizosphere soil physicochemical properties and microbial community structure exhibited strong spatial heterogeneity and host specificity, with *N. roborowskii* having a more complex microbial network and *A. sparsifolia* showing higher modularity. Multivariate factor analysis elucidated couplings among plant traits, soil properties, enzymes, and microbes. The two species form distinct interaction systems adapted to desert saline–alkali stress, advancing the understanding of ecological adaptation and informing restoration.

## Simple Summary

This study investigates how desert gradient heterogeneity affects plant–soil–microbe (PSM) systems of two xerophytic halophytes, *Alhagi sparsifolia* and *Nitraria roborowskii*, in Xinjiang’s Ebinur Lake wetland. *A. sparsifolia* uses a fast-growth strategy with a plastic crown area, larger height, and higher leaf/root nitrogen, supported by enhanced rhizospheric organic matter decomposition and nitrogen-cycling enzymes. *N. roborowskii* adopts a conservative strategy with stable crowns, adjusted phosphorus allocation, and optimized phosphorus cycling via rhizospheric properties. Rhizospheric microbes are host-specific: *N. roborowskii* has enriched Cyanobacteria, halotolerant archaea, and a complex network; *A. sparsifolia* has more viruses and a modular network. These findings reveal distinct adaptive strategies of the two species in forming unique PSM interaction systems to cope with desert saline–alkali stress, providing insights for ecological restoration of saline–alkali lands.

## 1. Introduction

Desert ecosystems, characterized by extreme aridity, drastic temperature fluctuations, and nutrient-poor, heterogeneous soils, pose severe challenges to plant survival, which relies heavily on intricate interactions with rhizosphere microbes and soil environments [1]. Traditional studies often focused on single factors, but advances in high-throughput sequencing and network analysis now enable the dissection of the dynamic equilibrium of plant–microbe–soil coupling systems from broader perspectives [2,3].

The rhizosphere, a critical hub for material exchange and energy flow at the soil–plant interface [4], mediates these interactions: plants release up to 40% of photosynthate as root exudates (e.g., sugars, amino acids) to fuel microbial communities [5,6], while rhizobacteria (e.g., PGPR) enhance plant growth via nutrient mobilization, phytohormone secretion, and stress tolerance [7,8,9,10]. For instance, specific bacterial genera (*Massilia*, *Burkholderia*) affect legume biomass, with microbial metabolism (e.g., terpenoid synthesis) correlating positively with plant productivity [1]. Desert spatial heterogeneity—manifested in water availability, nutrient distribution, and soil physicochemical properties (e.g., pH, salinity)—further modulates these interactions [11,12]. Soil physicochemical properties are widely recognized as dominant factors shaping the structure and function of rhizosphere microbial communities [4,13]. For example, pH, as one of the most critical soil properties, is widely regarded as a key driver of microbial community composition and diversity [14,15]. In the extreme heterogeneity of deserts, even minor differences in soil properties between adjacent areas may lead to significant divergence in rhizosphere microbial community structure, thereby altering their growth-promoting effects on xerophytic plants.

Furthermore, the intrinsic traits of plant species also play a pivotal role in shaping rhizosphere microbial communities. Different plant species, or even the same plants at different growth stages, selectively recruit and shape rhizosphere microbial communities through variations in the quality and quantity of root exudates [12,16]. In desert ecosystems, xerophytic plants have evolved distinct physiological and ecological strategies to cope with extreme aridity, such as developing deeper root systems, higher water-use efficiency, and specialized root exudation patterns to acquire water and nutrients [17]. These adaptive mechanisms are closely associated with the assembly and functionality of their rhizosphere microbial communities. Research by Jiang et al. on flue-cured tobacco rhizosphere bacterial communities revealed that bacterial assemblages exhibited clear habitat specificity across different ecoregions (representing varying habitat conditions), with community similarity significantly declining with increasing spatial distance. This further highlights the importance of habitat heterogeneity in rhizosphere microbial assembly [18]. The study also identified soil pH, available iron, exchangeable magnesium, and available manganese as key factors influencing rhizosphere bacterial structure. Notably, certain core microbes (e.g., *Micromonospora*, *Bryobacter*, *Arenimonas*) played critical roles in maintaining microbial network stability, though their importance was not solely determined by relative abundance but likely influenced by interactions with other microbial taxa and environmental conditions [18]. These findings provide valuable insights into the complexity of plant–microbe–soil interactions in desert environments.

Desert spatial heterogeneity not only directly affects soil physicochemical properties but also indirectly drives the assembly and functional diversity of rhizosphere microbial communities by altering the growth performance and root exudation patterns of xerophytic plants [19]. For instance, in microhabitats with relatively favorable moisture conditions, xerophytic plants may exhibit more vigorous growth, releasing a greater diversity and quantity of root exudates that attract a higher abundance of plant growth-promoting rhizobacteria (PGPR) [20]. Conversely, in extremely arid zones, limited plant growth and reduced root exudation may lead to decreased microbial diversity or favor the dominance of stress-tolerant microbial taxa [21]. Such cascading effects, triggered by environmental heterogeneity, could ultimately result in significant divergence in plant–microbe–soil interactions across different desert microhabitats, thereby influencing the overall functionality and stability of desert ecosystems.

Although existing studies have preliminarily revealed the influence of soil properties and plant species on rhizosphere microbial communities [1,4,13], the mechanisms by which gradient heterogeneity in deserts shapes these complex interactions and further affects the adaptive strategies of xerophytic plants remain poorly understood. For example, how fine-scale physicochemical variations in desert microhabitats (e.g., under shrubs, between dunes, different slopes) shape the composition of functional groups within rhizosphere microbial communities [22], how these functional groups feedback to plant growth and stress resistance, and how microbial co-occurrence networks exhibit distinct structural features and stability across heterogeneous microhabitats are still open questions. In response to these gaps, this study selects the xerophytic plants *A. sparsifolia* and *N. roborowskii* to investigate (i) how gradient-induced heterogeneity in soil properties alters epigeal–hypogeal functional trait relationships in xerophytic plants; (ii) how these changes further shape rhizosphere microbial community composition, structure, co-occurrence networks, and plant–microbe–soil interactions; and (iii) how these processes ultimately influence the growth and adaptation strategies of xerophytic plants. The findings are expected to shed new light on the ecological mechanisms driving the formation and persistence of biogeographic patterns in desert ecosystems.

## 2. Materials and Methods

### 2.1. Study Areas and Experimental Design

**Figure 1:**
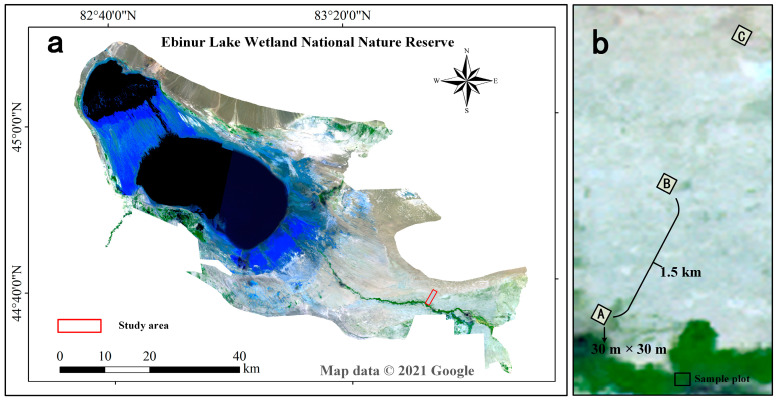
Overview map of the study area. Ebinur Lake Basin (**a**) and the sample plots. (**b**) Plot A, Plot B, and Plot C represent riverain forest, harsh desert, and desert margin, respectively.

The study area, located in the Ebinur Lake Wetland National Nature Reserve (44°30′–45°09′ N, 82°36′–83°50′ E) within the Bortala Mongol Autonomous Prefecture of Xinjiang, covers a total area of 2670.85 km^2^ (Figure 1a) [23]. The region features a typical desert–oasis transitional landscape and exhibits an arid climate with scarce precipitation, characteristic of a typical temperate continental arid climate. Extreme maximum temperatures can exceed 44 °C, while minimum temperatures may fall to −34 °C, with an annual average temperature of approximately 5 °C. Mean annual precipitation is about 105.17 mm, and mean annual evaporation reaches approximately 1315 mm [24]. Influenced by topography and climate, the soils are highly saline and alkaline. Salinity levels (measured as electrical conductivity, EC) across the experimental sites range from 4.49 to 8.09 μS cm^−1^; alkalinity (indicated by soil pH) varies from 7.40 to 8.28. The vegetation displays a distinct transitional pattern shaped by the overlapping influences of Central Asian and Mongolian floristic regions, making it one of the most species-rich desert plant areas in Xinjiang. Xerophytic desert plants, including small trees, shrubs, and semi-shrubs, dominate the landscape [25]. Plant and soil samples were collected near the Dongdaqiao Management Station, northeast of the Aqikesu River. A 3 km transect was established perpendicular to the riparian forest. Along this transect, three 30 m × 30 m quadrats (spaced approximately 1.5 km apart) were selected at equal intervals to capture the natural succession gradient. Quadrats A, B, and C represent the riparian forest, desert, and desert margin, respectively (Figure 1b).

### 2.2. Plant Collection

Sampling was conducted during non-precipitation periods under clear sky or low-cloud cover conditions. Within Plots A–C, healthy *A. sparsifolia* and *N. roborowskii* plants (typical xerophytic deep-rooted shrubs and dominant species in the Ebinur Lake desert) with similar growth statuses were selected. For each plant species within each plot, a total of nine individual plants were sampled.

Field measurements included recording morphological parameters for each plant: plant height (H) and crown area (CA). Trait measurements followed standardized protocols. Using pole pruners, four well-developed branches were collected from each plant canopy (from center to periphery along the four cardinal directions). From each branch, five fresh, intact mature leaves and current-year twigs (mass ≥ 10 g, diameter 1–2 cm) were collected. Stem length was measured from the base to the tip of the current-year twig using a measuring tape. For hypogeal sampling, considering that root biomass is primarily distributed within the upper 30 cm soil layer, a cylindrical sampling area (30 cm radius × 30 cm depth) was established. Woody roots (1–2 cm diameter) within 30 cm of the main stem base were excavated to minimize tissue tension effects. Leaves and roots were separately stored in ziplock bags for subsequent laboratory analysis. Plant samples were analyzed for specific leaf area (SLA), leaf carbon content (LCC), leaf nitrogen content (LNC), leaf phosphorus content (LPC), leaf dry matter content (LDMC), root dry matter content (RDMC), root carbon content (RCC), root nitrogen content (RNC), and root phosphorus content (RPC) (Table S1) [26].

### 2.3. Soil Collection

Soil samples were collected from the same depth as root samples (0–30 cm). Three subsamples were obtained per soil layer: the first was stored in aluminum boxes for determination of soil relative water content (SWC); the second was placed in sealed bags for analysis of physicochemical properties and enzyme activities; the third portion involved carefully excavating plant roots and gently removing loosely adhered soil while retaining the tightly bound rhizosphere soil, which was then placed in sterile bags and stored at 4 °C for subsequent metagenomic analysis of rhizosphere microbial communities. Soil samples from three plants were thoroughly mixed to form one composite sample, resulting in three composite samples (replicates) per species per plot (3 plots × 2 plant species × 3 replicates = 18 composite samples).

Soil analyses included pH, electrical conductivity (EC), soil organic carbon (SOC), total nitrogen (STN), and total phosphorus (STP). Detailed methodologies are provided in Table S1. Activities of alkaline phosphatase (ALP), *L*-leucine aminopeptidase (LAP), *β*-1,4-N acetylglucosaminidase (NAG), and *β*-1,4-glucosidase (BG) were determined using 96-well microplate assays based on α-naphthylamine colorimetry [27]. Enzyme activities were measured using commercial assay kits (Comin Biotechnology Co., Suzhou, China) with fluorescence detection by a Spark^TM^ Multimode Microplate Reader (Tecan, Männedorf, Switzerland).

### 2.4. DNA Extraction and Amplicon Sequencing

All pretreatment procedures were performed under sterile conditions in a Class II laminar flow hood. Immediately after collection, each root sample was placed in a sterile 50 mL conical tube and vortexed with 30 mL of sterile PBS buffer (137 mmol L^−1^ NaCl, 2.7 mmol L^−1^ KCl, 8.5 mmol L^−1^ Na_2_HPO_4_, 1.5 mmol L^−1^ KH_2_PO_4_, pH 7.3) (Thermo Fisher Scientific, Waltham, MA, USA) to dislodge rhizosphere-attached microorganisms. After removing the roots with sterilized forceps, the resulting suspension was filtered through sterile gauze into new 50 mL sterile centrifuge tubes. The filtrate was centrifuged at 10,000× *g* for 1 min at 4 °C. The supernatant was carefully discarded and the microbial pellet was stored at −80 °C for downstream analysis. Total genomic DNA was extracted from 0.2 g of rhizosphere soil using the E.Z.N.A.^®^ Soil DNA Kit (OMEGA Bio-Tek, Norcross, GA, USA) following the manufacturer’s protocol. DNA integrity was verified by 1% agarose gel electrophoresis (200 V, 30 min) and quantified using both Qubit 2.0 fluorometer and NanoDrop ND-2000 spectrophotometer (NanoDrop Technologies, Wilmington, DE, USA).

Sequence data processing was performed as follows: Raw reads were quality-filtered using Trimmomatic (v0.39) to remove adapter sequences and low-quality bases (Phred score < 20), retaining reads with length > 150 bp. Paired-end reads were merged using FLASH (v1.2.11) with a minimum overlap of 10 bp. Amplicon Sequence Variants (ASVs) were clustered using DADA2 (v1.20) to resolve sequence variants with 100% identity, and chimeric sequences were removed using the “consensus” method. Taxonomic assignment of ASVs was performed against the SILVA database (v138) for prokaryotes and UNITE database (v8.2) for fungi using BLASTn, with a minimum identity threshold of 97%. Paired-end libraries were constructed and sequenced on an Illumina NovaSeq platform (PE150, Illumina Inc., San Diego, CA, USA) at Sangon Biotech (Shanghai, China), generating 5–10 Gbp raw data per sample. All raw sequences were deposited in the NCBI Sequence Read Archive under accession number PRJNA664310.

### 2.5. Statistical Analysis

Gradient differences in functional traits were analyzed using one-way ANOVA, with results expressed as Mean ± SD (n = 3). Mantel tests were employed to examine relationships between microbial community structure and soil physicochemical parameters. Prior to microbial network analysis, ASVs that were absent in at least one replicate and had a total relative abundance < 0.01% across all samples were filtered out. Microbial co-occurrence patterns were inferred based on Spearman correlations (*r* ≥ 0.6, FDR-adjusted *p* < 0.05, n = 9), and microbial networks along with relevant topological properties were computed using the igraph package in R (version 4.2.1 Development Core Team; https://www.r-project.org/). Microbial networks were visualized using Gephi software (version 0.10.1). Relationships between biotic factors (plant functional traits and microbial communities) and abiotic factors (e.g., soil physicochemical properties) were analyzed using the cor function in the vegan and ggcor packages for R version 4.2.1. Multivariate factor analysis was conducted to assess correlations among plant traits, microbial communities, and soil physicochemical parameters, implemented via the FactoMineR package.

## 3. Results

### 3.1. Plant Functional Traits

One-way ANOVA revealed significant interspecific and gradient-dependent variations in functional traits (Table 1). *A. sparsifolia* showed marked differences in crown area (CA) across plots, with Plot A values significantly higher than Plots B and C (*p* < 0.05). In contrast, *N. roborowskii* maintained a stable CA but exhibited significant variations in leaf phosphorus (LPC) and root phosphorus (RPC) across plots, with lower values in Plots B and C than Plot A. Trait correlation patterns differed: *A. sparsifolia* showed weak epigeal–hypogeal coordination (Figure S1a), while *N. roborowskii* displayed hypogeal trait synergies and epigeal–hypogeal nutrient trade-offs, indicating divergent resource allocation strategies (Figure S1b).

### 3.2. Soil Environmental Factors

Rhizosphere soil properties and enzyme activities exhibited distinct spatial gradients (Table 2). From Plot A to C, soil pH, water content (SWC), electrical conductivity (EC), and nutrient concentrations (e.g., STN, STP) decreased monotonically. EC showed species-specific differences in Plots B and C, with higher values under *N. roborowskii* than *A. sparsifolia*. Most enzyme activities varied significantly across habitats, with limited interspecific differences (only in NAG, STN, and STP in specific plots), indicating gradient heterogeneity as the primary driver.

### 3.3. Rhizosphere Soil Microorganisms

**Figure 2:**
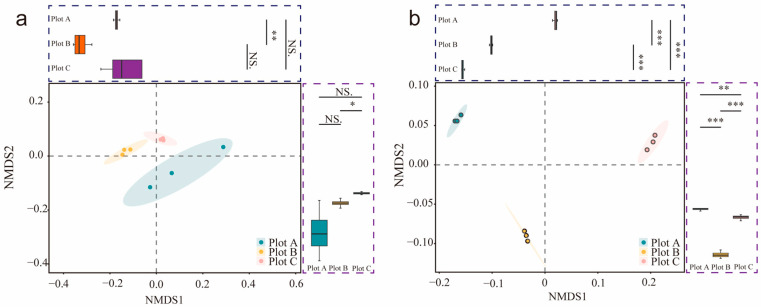
Microbial community structure. NMDS analysis of *A. sparsifolia* (**a**) and *N. roborowskii* (**b**). * *p* < 0.05, ** *p* < 0.01, *** *p* < 0.001, “NS” indicates non–significance.

**Figure 3:**
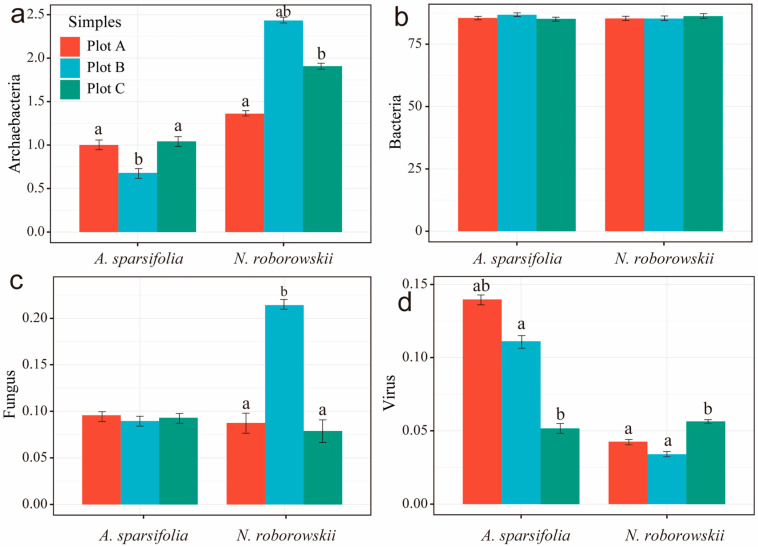
Difference in relative abundance of microorganisms (**a**–**d**) of *A. sparsifolia* and *N. roborowskii*. The same lowercase letters indicate no significant difference among different plots (*p* > 0.05), and different lowercase letters indicate a significant difference among different plots (*p* < 0.05).

**Figure 4:**
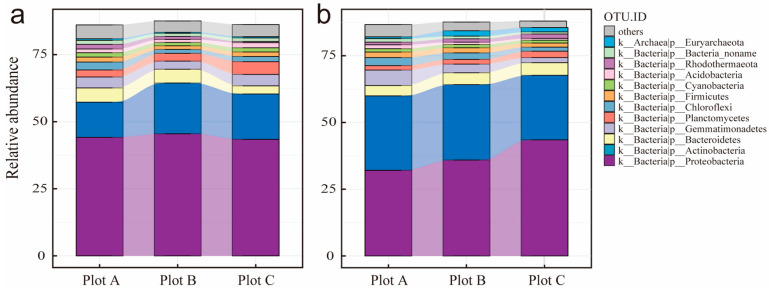
The relative abundance of microbial phylum level (top 0.01%) of *A. sparsifolia* (**a**) and *N. roborowskii* (**b**).

The spatial distribution of rhizosphere soil microorganisms differed significantly between the two plant species, *A. sparsifolia* and *N. roborowskii*, as evidenced by distinct microbial community compositions (Figure 2). The microbial communities of the two species exhibited clear gradient variations and interspecific divergence across sampling plots (Figure 3). *N. roborowskii* rhizospheres were enriched in archaea (e.g., *Halalkalicoccus*, *Halorubrum*) and Cyanobacteria, while *A. sparsifolia* had higher viral abundance (e.g., *Salterprovirus*, *Siphoviridae*) (Figure 4 and Figure S2). Both shared dominant phyla (Proteobacteria, Actinobacteria), but *A. sparsifolia* had more Acidobacteria (12.4%), and *N. roborowskii* more Cyanobacteria (9.8%). Phylogenetic tree analysis of microbial abundance confirmed that bacterial and viral nodes had higher relative abundance in *N. roborowskii*, whereas archaeal and viral nodes were less abundant in *N. roborowskii* compared to *A. sparsifolia* (Figure S3).

**Figure 5:**
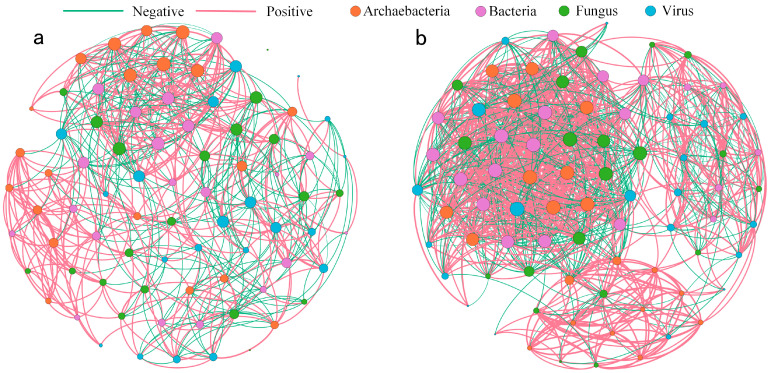
Rhizosphere microbial co-occurrence networks at genus level in *A. sparsifolia* (**a**) and *N. roborowskii* (**b**).

**Figure 6:**
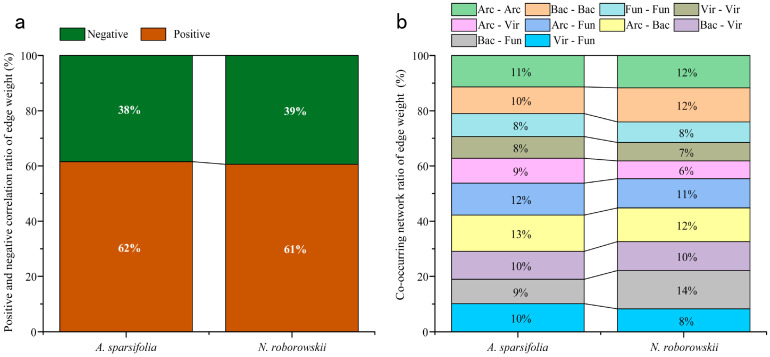
Network edge properties in *A. sparsifolia* (**a**) and *N. roborowskii* (**b**).

**Figure 7:**
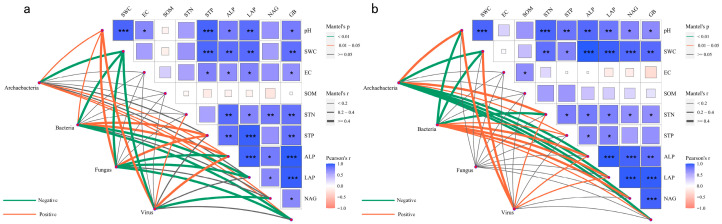
Mantel test correlations between soil indicators and rhizosphere microorganisms in *A. sparsifolia* (**a**) and *N. roborowskii* (**b**). * *p* < 0.05, ** *p* < 0.01, *** *p* < 0.001.

The co-occurrence networks differed structurally (Figure 5): *N. roborowskii* had a more complex, tightly connected network (higher edge number, density, and clustering coefficient), while *A. sparsifolia* showed higher modularity and longer path lengths, indicating functional compartmentalization (Figure 6, Table 3). Mantel tests linked soil factors to microbial communities: in *A. sparsifolia*, archaeal, bacterial, fungal, and viral communities correlated with pH, STP, and enzyme activities (e.g., ALP, BG); in *N. roborowskii*, archaea correlated with pH and nutrients, while bacteria correlated with enzyme activities, reflecting species-specific response patterns (Figure 7).

### 3.4. Relationships Among Plant Functional Traits, Rhizosphere Soil Physicochemical Properties, Enzyme Activities, and Microorganisms

**Figure 8:**
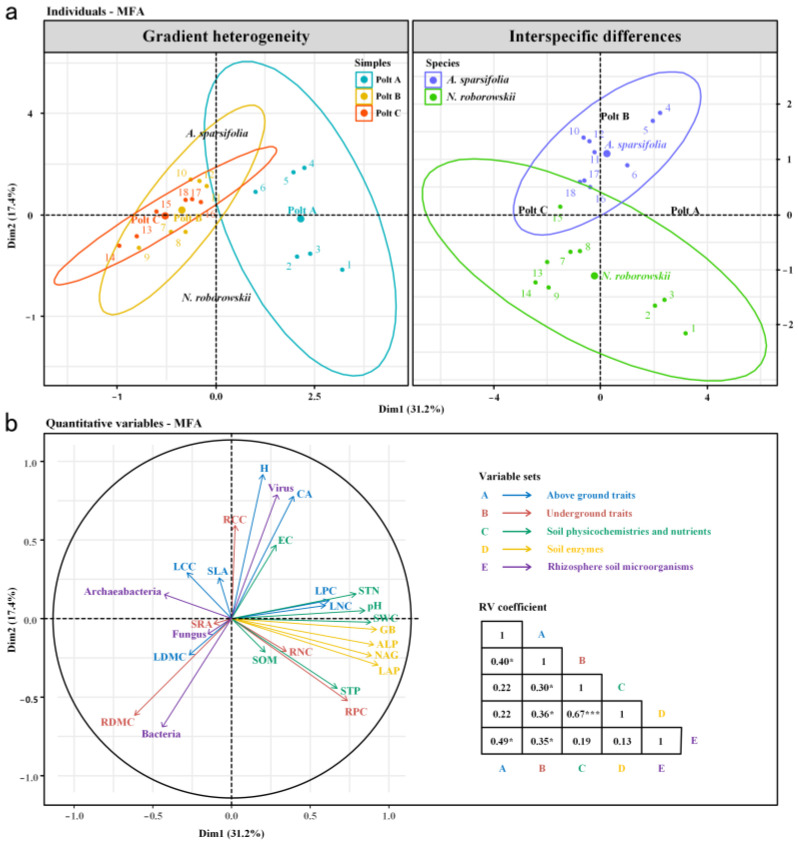
Multivariate factor analysis (MFA) of plant functional traits, rhizosphere soil physicochemical properties, enzyme activities, and microorganisms in *A. sparsifolia* and *N. roborowskii*. The individual map of MFA (**a**). The quantitative variable map of MFA (**b**). * *p* < 0.05, *** *p* < 0.001.

Multivariate factor analysis (MFA) revealed clear differentiation between species in PSM systems (Figure 8, Table S2). *A. sparsifolia* clustered in the positive region of Dimension 1 (dim1, explaining 31.2% of the total variance, driven by CA, H, LNC, RNC), while *N. roborowskii* clustered in the negative region (driven by RDMC, SLA). Dimension 2 (dim2, explaining 17.4% of variance) reflected soil properties, with SOM, STN, and enzyme activities (BG, NAG) positively correlated and pH, EC, and ALP negatively correlated.

**Figure 9:**
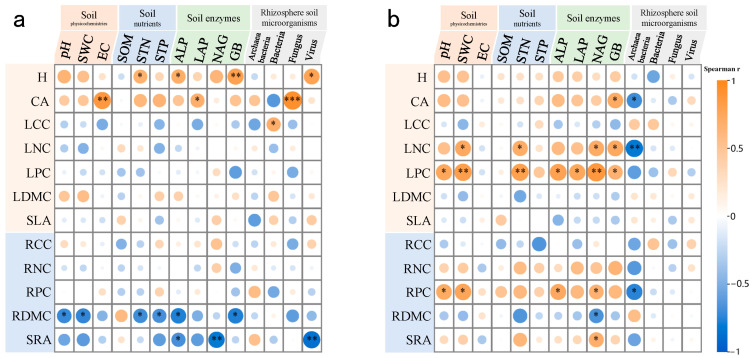
Spearman’s rank correlation coefficient of above- and underground functional traits with soil physicochemical properties, soil enzyme activity, and rhizosphere soil microorganisms. *A. sparsifolia* (**a**) and *N. roborowskii* (**b**). * *p* < 0.05, ** *p* < 0.01, *** *p* < 0.001.

Correlations between traits and soil/microbes differed: *A. sparsifolia* epigeal traits (H, CA) correlated with EC and fungal abundance (Figure 9a); *N. roborowskii* LPC correlated with SWC, STN, and NAG (Figure 9b), with archaeal abundance negatively linked to functional traits, indicating divergent microbiome assembly strategies.

## 4. Discussion

### 4.1. Gradient Heterogeneity of Functional Traits in Two Desert Halophytes

In desert ecosystems, plant functional trait responses to environmental heterogeneity are central to understanding their resource acquisition strategies and ecological adaptation capacity [28]. This study compared variation patterns of twelve functional traits between two typical desert halophytes, *A. sparsifolia* and *N. roborowskii*, across different plots, revealing their divergent responses to micro-environmental gradients in arid lands (Table 1). The results demonstrated significant inter-plot variations in crown area (CA) for *A. sparsifolia*, with Plot A showing higher values than Plots B and C (*p* < 0.05), suggesting a sensitive allocation of epigeal biomass in response to habitat conditions—likely influenced by variations in soil moisture and salinity. This observation aligns with findings by Zhao et al. reporting similar crown size variations in *Sophora alopecuroides* [29]. In contrast, *N. roborowskii* exhibited no significant CA differences across plots, suggesting either greater canopy structural plasticity or more conservative growth strategies toward local environmental fluctuations [30]. Regarding nutrient-related traits, *N. roborowskii* showed significant plot-level variations in both LPC and RPC, with Plots B and C displaying lower values than Plot A (*p* < 0.05), reflecting high responsiveness to environmental phosphorus availability. This is consistent with the research findings of García-Palacios et al., which demonstrate that plant nutrient content is highly sensitive to soil heterogeneity in arid ecosystems [31]. Conversely, *A. sparsifolia* maintained stable LPC and RPC across plots, indicating possible homeostasis through consistent nutrient acquisition mechanisms [32].

Trait correlation analyses further revealed interspecific divergence in resource allocation strategies (Figure S1). The lack of epigeal–hypogeal trait coordination in *A. sparsifolia* suggests organ-specific functional specialization—enhancing photosynthetic efficiency (epigeal) while maintaining basic absorptive functions (hypogeal), a pattern prevalent among halophytes [33]. *N. roborowskii*, however, exhibited hypogeal trait synergies and epigeal–hypogeal nutrient trade-offs, demonstrating whole-plant resource optimization under limiting conditions [34]. While most traits remained stable across plots, the sensitivity of *A. sparsifolia*’s CA and *N. roborowskii*’s LPC/RPC to habitat heterogeneity highlights their potential as environmental response indicators [35]. Such gradient-dependent trait variation reflects species-specific adaptation pathways to abiotic stress, facilitating functional complementarity and niche differentiation that support species coexistence [36]. The two halophytes exhibited distinct adaptive mechanisms along micro-environmental gradients: *A. sparsifolia* primarily modulates epigeal traits, whereas *N. roborowskii* optimizes resource use through nutrient allocation adjustments. These differential strategies represent ecological trade-offs between functional stability and adaptive plasticity [37], providing theoretical frameworks for understanding structure–function relationships in desert plant communities.

### 4.2. Gradient Heterogeneity of Soil Properties and Rhizosphere Microbial Community Structure in Two Desert Halophytes

In extreme environments, plant–rhizosphere microbe interactions play a pivotal role in maintaining ecosystem stability. Within desert ecosystems, saline–alkali stress significantly constrains plant survival and reproduction [38]. Our investigations on *A. sparsifolia* and *N. roborowskii* revealed distinct spatial gradients in rhizosphere soil physicochemical properties and microbial community structure, characterized by monotonic decreases in pH, SWC, EC, and nutrient concentrations from Plot A to Plot C (Table 2), demonstrating that gradient heterogeneity serves as the primary driver of soil environmental variation. These findings align with previous studies confirming the substantial impact of microhabitat differences on soil characteristics in arid saline–alkali environments [39]. By contrast, plant species exerted relatively weaker influences on soil physicochemical properties, with interspecific differences only observed in specific parameters including EC, STN, STP, and particular enzyme activities (e.g., NAG). This suggests that in extreme environments, plants exhibit limited capacity for soil feedback regulation, with macroenvironmental factors playing a dominant role [40].

Conversely, rhizosphere microbial community structure displayed pronounced differentiation between plant species (Figure 2, Figure 3 and Figure 4). NMDS analysis and community composition statistics demonstrated significant interspecific divergence in four major microbial groups (bacteria, fungi, archaea, and viruses) between *N. roborowskii* and *A. sparsifolia* rhizospheres, with host plant effects surpassing plot-level variations (Figures S2 and S3). This underscores the central role of plant hosts in shaping microbial community assembly [41]. *N. roborowskii* rhizospheres were enriched with salt-tolerant microbial taxa including Cyanobacteria and halophilic archaeal genera (e.g., *Halalkalicoccus*, *Halorubrum*), reflecting adaptations to high-salinity conditions. In contrast, *A. sparsifolia* exhibited viral enrichment (e.g., *Siphoviridae*, *Myoviridae*), potentially associated with reduced rhizosphere microbial stability under saline–alkali stress [42].

Regarding co-occurrence network topology, *N. roborowskii* demonstrated microbial networks with higher connectivity and clustering coefficients (Figure 5 and Figure 6, Table 2), indicating more intensive inter-microbial interactions and greater network stability. This observation supports Luo et al.’s proposition that complex co-occurrence networks enhance microbial community resilience to environmental disturbances [43]. In contrast, the rhizosphere microbiome of *A. sparsifolia* displayed higher network modularity and a longer path length, suggesting enhanced functional compartmentalization and pronounced niche differentiation—evolutionary adaptations to microenvironmental instability [44]. Mantel tests further revealed significant correlations between soil factors and microbial communities (Figure 7). In *N. roborowskii* systems, archaeal communities showed positive correlations with pH, STN, and STP; bacterial communities correlated positively with soil enzyme activities; fungal communities displayed no clear soil factor associations; and viral communities primarily correlated with pH and nutrients. These patterns demonstrate distinct response mechanisms among microbial groups to soil physicochemical factors, with response patterns being plant-species dependent [45]. The *A. sparsifolia* system exhibited more complex microbe–soil factor relationships, reflecting higher systemic instability.

Notably, plant regulation of microbial communities extends beyond compositional assembly to include functional network construction (Table 2). *N. roborowskii*’s more interconnected microbial network suggests enhanced capacity to recruit functionally complementary microbes under salt stress, thereby improving stress tolerance [46]. *A. sparsifolia*’s modular network architecture may instead rely on distinct microbial modules performing specialized functions, buffering against systemic risks from community fluctuations. Despite inhabiting identical desert saline–alkali environments, the two halophytes maintained significantly divergent rhizosphere soil properties and microbial communities. This differentiation was primarily governed by plant species identity, while soil characteristics were more strongly controlled by gradient heterogeneity. Through root exudation, nutrient release, and pH modulation, plants critically influence rhizosphere microbiomes [47]. Our results reinforce the dominant role of host plants in structuring microbial communities, particularly pronounced in extreme ecosystems.

### 4.3. Interactions Between Functional Traits, Soil Properties, and Rhizosphere Microbial Structure in Two Desert Halophytes

Multiple factor analysis (MFA) elucidated the ecological coupling processes in plant–soil–microbe systems by revealing significant differences and interrelationships in functional traits, rhizosphere soil properties, and microbial communities between the desert halophytes *A. sparsifolia* and *N. roborowskii* (Figure 8). The analysis demonstrated clear differentiation between the two species along both functional trait (Dimension 1) and soil property (Dimension 2) axes, reflecting fundamental divergence in their ecological adaptation strategies. *A. sparsifolia* exhibited a larger crown area, greater plant height, and higher leaf and RNC, suggesting a resource-acquisitive growth strategy. These traits showed positive correlations with SRA and RCC, indicating coordinated resource allocation [48]. In contrast, *N. roborowskii* displayed higher RDMC and SLA, representing a more conservative growth strategy to enhance tolerance under drought and saline–alkali stress [49]. These functional trait differences reflect distinct physio-ecological adaptation pathways in desert environments, consistent with previous findings on plant functional trait diversity under drought stress [50,51]. The rhizosphere environments differed substantially between species. *A. sparsifolia* maintained higher SOM and STN, along with elevated activities of *β*-1,4-glucosidase and *β*-1,4-N-acetylglucosaminidase, indicating enhanced carbon and nitrogen cycling [52]. *N. roborowskii* rhizospheres showed higher pH, EC, and alkaline phosphatase activity, reflecting adaptation to saline conditions and phosphorus mineralization [53]. SWC correlated significantly with phosphorus-related traits in *N. roborowskii*, demonstrating hydrological linkages in nutrient acquisition. SWC was significantly correlated with LPC and RPC in *N. roborowskii*, further indicating that this species may adapt to saline–alkali stress by regulating phosphorus utilization. This finding aligns with the documented mechanism whereby halophytes enhance nutrient availability through rhizosphere soil modification under salt stress [54]. The rhizosphere microbial community structure showed close correlations with plant functional traits (Figure 9). In *A. sparsifolia*, epigeal traits (H and CA) were significantly correlated with soil microbial groups and electrical conductivity (EC), while specific root area (SRA) exhibited significant associations with fungal and viral communities. These relationships indicate that the rhizosphere microbiome plays important roles in promoting plant growth and stress resistance in this species [4]. By contrast, in *N. roborowskii*, LPC showed significant correlations with SWC, STN, and NAG activity, suggesting that this species optimizes resource utilization through regulation of rhizosphere nitrogen–phosphorus cycling and microbial interactions [55]. Additionally, soil archaea displayed negative correlations with functional traits in both plant species, potentially reflecting either inhibitory effects of these microorganisms on plants or niche competition under saline–alkali stress conditions [56]. These findings are consistent with recent research trends demonstrating that plant functional traits regulate rhizosphere microbial community structure, which in turn feeds back to influence plant growth [57,58].

In summary, the two halophyte species employ distinct functional trait strategies to modulate rhizosphere soil environments and microbial community structure, thereby forming synergistic ecological networks to adapt to desert saline–alkali stress. *A. sparsifolia* tends to establish a rhizosphere microbial environment that facilitates organic matter decomposition and nitrogen cycling to promote rapid growth, while *N. roborowskii* enhances stress tolerance by regulating rhizosphere saline–alkali conditions and optimizing phosphorus cycling. This study provides an empirical foundation for understanding plant–soil–microbe interactions in saline–alkali deserts and highlights functional traits as key drivers of rhizosphere ecosystem processes. Future research should further investigate the causal relationships between functional traits and microbial functional diversity, and their impacts on the stability and resilience of desert ecosystems, which will facilitate ecological restoration and sustainable management of saline–alkali lands.

## 5. Conclusions

This study systematically explored the interactions between functional traits, rhizosphere soil properties, and microbial communities of two desert halophytes, *A. sparsifolia* and *N. roborowskii*, across environmental gradients in Xinjiang’s Ebinur Lake wetland. The findings reveal distinct adaptive strategies: *A. sparsifolia* adopts a fast-growth strategy, with plastic crown development and efficient nitrogen use supported by rhizospheric nitrogen-cycling enzymes and organic matter decomposition, while *N. roborowskii* employs a conservative strategy, maintaining stable crowns and optimizing phosphorus allocation via rhizospheric regulation and phosphorus-cycling enzymes. Rhizosphere microbial communities further reflect host specificity: *N. roborowskii* harbors Cyanobacteria and halotolerant archaea within complex, stable networks, enhancing stress resistance, whereas *A. sparsifolia* exhibits higher viral abundance and modular microbial networks, adapting to microenvironmental fluctuations. These differences underscore how desert gradient heterogeneity drives divergence in plant–soil–microbe systems, shaping unique adaptive strategies.

To advance ecological applications, future research should focus on (1) clarifying causal relationships between specific root exudates, microbial functional groups (e.g., phosphorus-mobilizing bacteria), and plant trait expression, to inform targeted microbial inoculation for restoration; (2) scaling these findings to guide species selection—e.g., using *A. sparsifolia* for nitrogen-poor but moderately saline zones to accelerate vegetation establishment, and *N. roborowskii* for extreme saline–alkali sites to stabilize soil via its robust microbial associations; and (3) exploring how these adaptive strategies influence ecosystem functions (e.g., carbon sequestration) across broader desert gradients. Such insights will enhance the precision of saline–alkali land restoration and sustainable management practices.

## Figures and Tables

**Table 1:** One-way analysis of variance for plant functional traits (*p* < 0.05), with data presented as mean ± SE. Plant height (H), crown area (CA), leaf carbon content (LCC), leaf nitrogen content (LNC), leaf phosphorus content (LPC), leaf dry matter content (LDMC), specific leaf area (SLA), root carbon content (RCC), root nitrogen content (RNC), root phosphorus content (RPC), root dry matter content (RDMC), specific root area (SRA). The same lowercase letters indicate no significant difference among different plots (*p* > 0.05), and different lowercase letters indicate a significant difference among different plots (*p* < 0.05).

Samples	Variable	*A. sparsifolia*	*N. roborowskii*	Variable	*A. sparsifolia*	*N. roborowskii*
Plot A	H (cm)	74.33 ± 6.63 a	0.50 ± 0.09 a	SLA (cm^2^ g^−1^)	113.44 ± 30.83 a	95.46 ± 43.24 a
Plot B		69.33 ± 4.67 a	0.38 ± 0.01 a		141.69 ± 10.77 a	106.48 ± 13.69 a
Plot C		59.00 ± 2.65 a	0.35 ± 0.02 a		123.51 ± 6.24 a	121.38 ± 9.93 a
Plot A	CA (cm^2^)	4448.50 ± 683.64 a	3.19 ± 1.90 a	RCC (g kg^−1^)	256.15 ± 15.67 a	238.36 ± 9.05 a
Plot B		1271.04 ± 204.36 b	0.24 ± 0.01 a		264.46 ± 5.84 a	245.73 ± 2.05 a
Plot C		1975.54 ± 265.13 b	0.56 ± 0.30 a		239.24 ± 5.00 a	16.97 ± 3.81 a
Plot A	LCC (g kg^−1^)	380.86 ± 4.72 a	374.93 ± 11.17 a	RNC (g kg^−1^)	13.304 ± 2.39 a	12.53 ± 0.86 a
Plot B		428.50 ± 19.86 a	391.34 ± 11.96 a		14.09 ± 2.17 a	14.42 ± 1.28 a
Plot C		385.62 ± 25.52 a	393.55 ± 16.56 a		14.31 ± 0.26 a	1.12 ± 1.57 a
Plot A	LNC (g kg^−1^)	20.309 ± 1.11 a	22.93 ± 0.88 a	RPC (g kg^−1^)	0.59 ± 0.05 a	0.46 ± 0.10 a
Plot B		19.08 ± 0.97 a	16.87 ± 0.74 b		0.50 ± 0.04 a	0.51 ± 0.07 b
Plot C		22.30 ± 0.53 a	17.66 ± 2.60 ab		0.57 ± 0.01 a	0.44 ± 0.13 b
Plot A	LPC (g kg^−1^)	1.083 ± 0.08 a	1.34 ± 0.13 a	RDMC (g g^−1^)	0.28 ± 0.00 b	0.69 ± 0.01 a
Plot B		1.23 ± 0.07 a	0.94 ± 0.08 b		0.41 ± 0.01 a	0.57 ± 0.05 a
Plot C		1.13 ± 0.05 a	0.84 ± 0.09 b		0.43 ± 0.03 a	6.43 ± 0.12 a
Plot A	LDMC (g g^−1^)	0.30 ± 0.01 a	0.32 ± 0.11 a	SRA (cm^2^ g^−1^)	3.34 ± 1.22 a	3.01 ± 0.47 a
Plot B		0.31 ± 0.01 a	0.32 ± 0.04 a		2.43 ± 0.12 a	13.13 ± 1.23 a
Plot C		0.28 ± 0.01 a	0.39 ± 0.12 a		7.79 ± 2.65 a	238.36 ± 11.20 a

**Table 2:** One-way analysis of variance of soil physicochemical properties, with data presented as mean ± SE. Soil pH, soil water content (SWC), electrical conductivity (EC). soil organic matter (SOM), soil total nitrogen (STN), soil total phosphorus (STP). Soil enzymes. Alkaline phosphatase (ALP), L-leucine aminopeptidase (LAP), *β*-1,4-N acetylglucosaminidase (NAG), *β*-1,4-glucosidase (BG). The same lowercase letters indicate no significant difference among different plots (*p* > 0.05), and different lowercase letters indicate a significant difference among different plots (*p* < 0.05).

Samples	Variable	*A. sparsifolia*	*N. roborowskii*	Variable	*A. sparsifolia*	*N. roborowskii*
Plot A	pH	8.28 ± 0.08 a	8.21 ± 0.10 a	STP (g kg^−1^)	0.84 ± 0.02 a	0.94 ± 0.11 a
Plot B		7.87 ± 0.02 b	7.89 ± 0.11 b		0.62 ± 0.03 b	0.78 ± 0.02 ab
Plot C		7.60 ± 0.09 c	7.40 ± 0.04 c		0.58 ± 0.03 b	0.65 ± 0.06 b
Plot A	SWC	15.92 ± 0.88 a	16.16 ± 0.20 a	ALP (nmol g^−1^ h^−1^)	4.92 ± 0.58 a	5.93 ± 0.54 a
Plot B		9.15 ± 0.48 b	8.05 ± 0.56 b		1.80 ± 0.02 b	2.33 ± 0.09 b
Plot C		3.75 ± 0.54 c	2.75 ± 0.34 c		1.24 ± 0.24 b	0.60 ± 0.05 c
Plot A	EC (μS cm^−1)^	8.09 ± 0.49 a	4.59 ± 0.44 a	LAP (nmol g^−1^ h^−1^)	14.91 ± 0.02 a	21.09 ± 0.58 a
Plot B		5.35 ± 0.47 b	6.06 ± 1.36 a		4.97 ± 0.02 b	5.84 ± 0.06 b
Plot C		5.76 ± 1.12 ab	4.49 ± 0.53 a		4.79 ± 0.17 b	3.58 ± 0.23 b
Plot A	SOM (g kg^−1^)	15.24 ± 2.32 a	18.37 ± 2.6 a	NAG (nmol g^−1^ h^−1^)	15.77 ± 4.61 a	26.32 ± 1.72 a
Plot B		15.07 ± 2.81 a	18.10 ± 4.25 a		8.57 ± 0.29 a	5.91 ± 1.37 b
Plot C		17.41 ± 3.65 a	12.56 ± 4.46 a		6.85 ± 0.06 a	5.94 ± 0.11 b
Plot A	STN (g kg^−1^)	0.99 ± 0.10 a	0.91 ± 0.05 a	GB (nmol g^−1^ h^−1^)	28.51 ± 2.8 a	30.53 ± 3.32 a
Plot B		0.73 ± 0.07 ab	0.76 ± 0.07 a		8.77 ± 1.95 b	5.39 ± 0.54 b
Plot C		0.66 ± 0.08 b	0.53 ± 0.07 b		7.83 ± 0.44 b	5.31 ± 0.31 b

**Table 3:** Topological properties of root-associated microbial co-occurrence networks of *A. sparsifolia* and *N. roborowskii*.

Properties	*A. sparsifolia*	*N. roborowskii*
Edge number	650	1176
Edge density	0.206	0.372
Diameter	6	4
Average path length	2.299	1.868
Modularity	2.497	1.865
Modules	7	3
Average degree	16.250	29.375
Clustering coefficient	0.646	0.786

## Data Availability

The original data presented in the study are included in the article; further inquiries can be directed to the corresponding author.

## Supplementary Materials

The following supporting information can be downloaded at: https://www.mdpi.com/article/10.3390/biology14081048/s1, Figure S1: Spearman correlation analysis between aboveground and belowground functional traits of Haloxylon ammodendron *A. sparsifolia* (a) and *N. roborowskii* (b). * *p* < 0.05, ** *p* < 0.01; Figure S2: T-test analysis of rhizosphere soil microorganisms at genus level: (a) Archaea, (b) Bacteria, (c) Fungi, (d) Viruses; Figure S3: Phylogenetic trees of rhizosphere microbial abundance at genus level: (a–d) *A. sparsifolia* (a) archaea, (b) bacteria, (c) fungi, (d) viruses; (e–h) *N. roborowskii* (e) archaea, (f) bacteria, (g) fungi, (h) viruses; Table S1: Measurement methods for soil and plant physicochemical properties; Table S2: Eigenvalues and cumulative interpretation rates from Multiple Factor Analysis.
