# Supplementary material for: The Gradient Heterogeneity of Deserts Alters the Interaction Relationships Between Xerophytic Plants and Soils
Source: Biology (Basel). 2025 Aug 14;14(8):1048. doi: 10.3390/biology14081048 (PMC12383998; doi:10.3390/biology14081048)
Supplement: Supplementary file 1 [file biology-14-01048-s001.zip › biology-3757130-supplementary.pdf]

Supplementary Materials:

The Gradient Heterogeneity of Deserts Alters the Interaction Relationships Between Xerophytic Plants and Soils

Jinlong Wang [1,2,3,*], Yudong Chen [1,2,3], Xiaotong Li [1,2,3], Xiaojuan Cao [1,2,3], Hongli Tang [1,2,3] and Guanghui Lv [1,2,3]

[1] College of Ecology and Environment, Xinjiang University, Urumqi 830017, China; ydaccepted@sina.com (Y.C.); lixiaotong@stu.xju.edu.cn (X.L.); cxj019085@163.com (X.C.); 17686206367@163.com (H.T.); ler@xju.edu.cn (G.L.)
[2] Key Laboratory of Oasis Ecology of Education Ministry, Xinjiang University, Urumqi 830017, China
[3] Xinjiang Jinghe Observation and Research Station of Temperate Desert Ecosystem, Ministry of Education, Jinghe 833300, China
* Correspondence: wangjl@xju.edu.cn

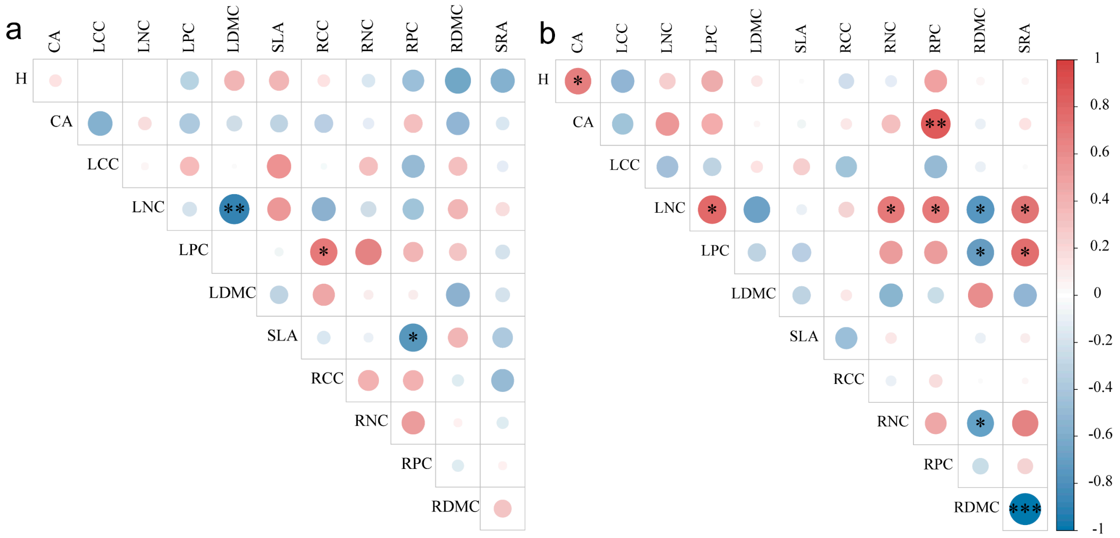

Figure S1. Spearman correlation analysis between aboveground and belowground functional traits of Haloxylon ammodendron *A. sparsifolia* (a) and *N. roborowskii* (b). *p < 0.05, **p < 0.01, ***p < 0.001.

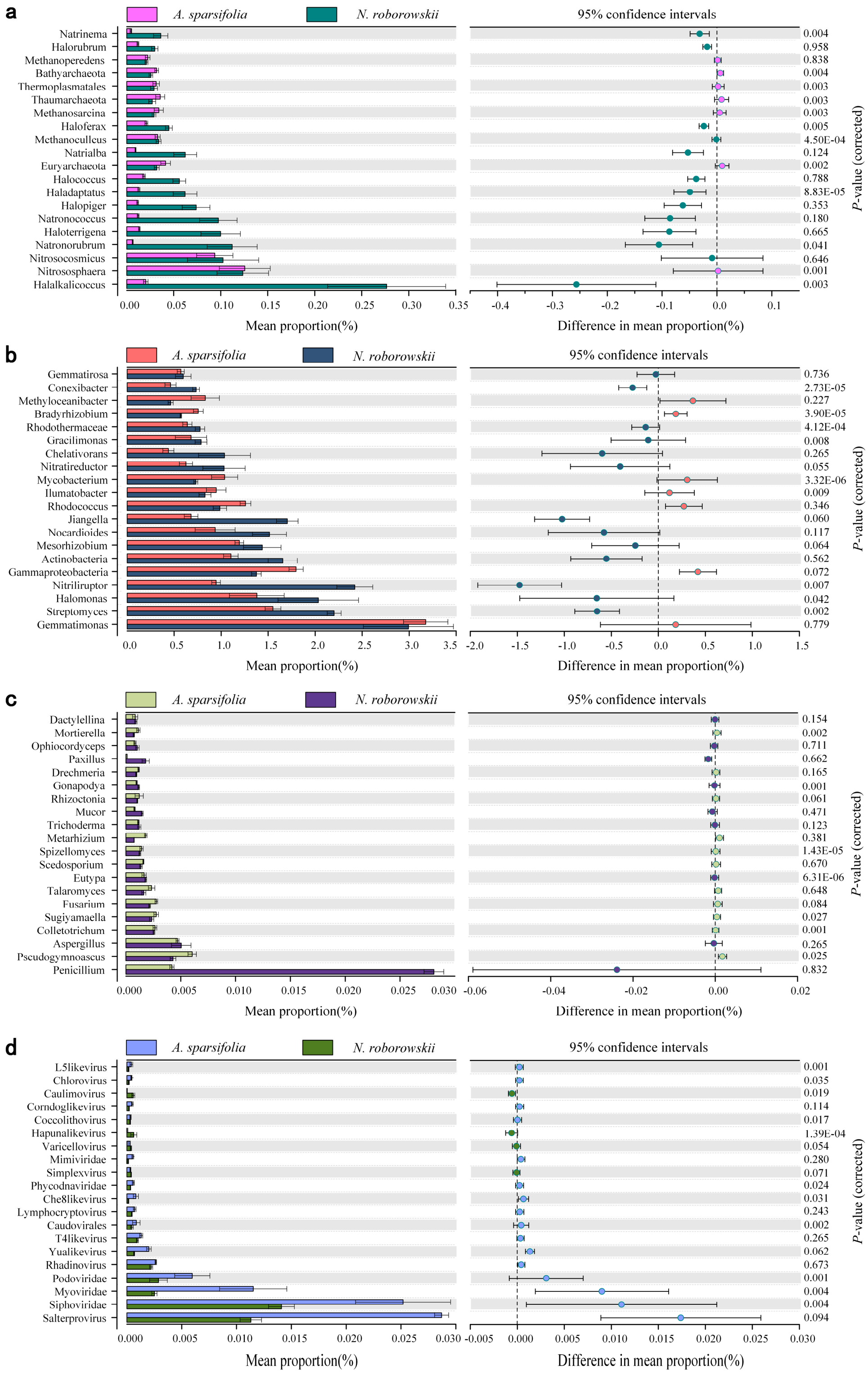

Figure S2. T-test analysis of rhizosphere soil microorganisms at genus level: (a) Archaea, (b) Bacteria, (c) Fungi, (d) Viruses.

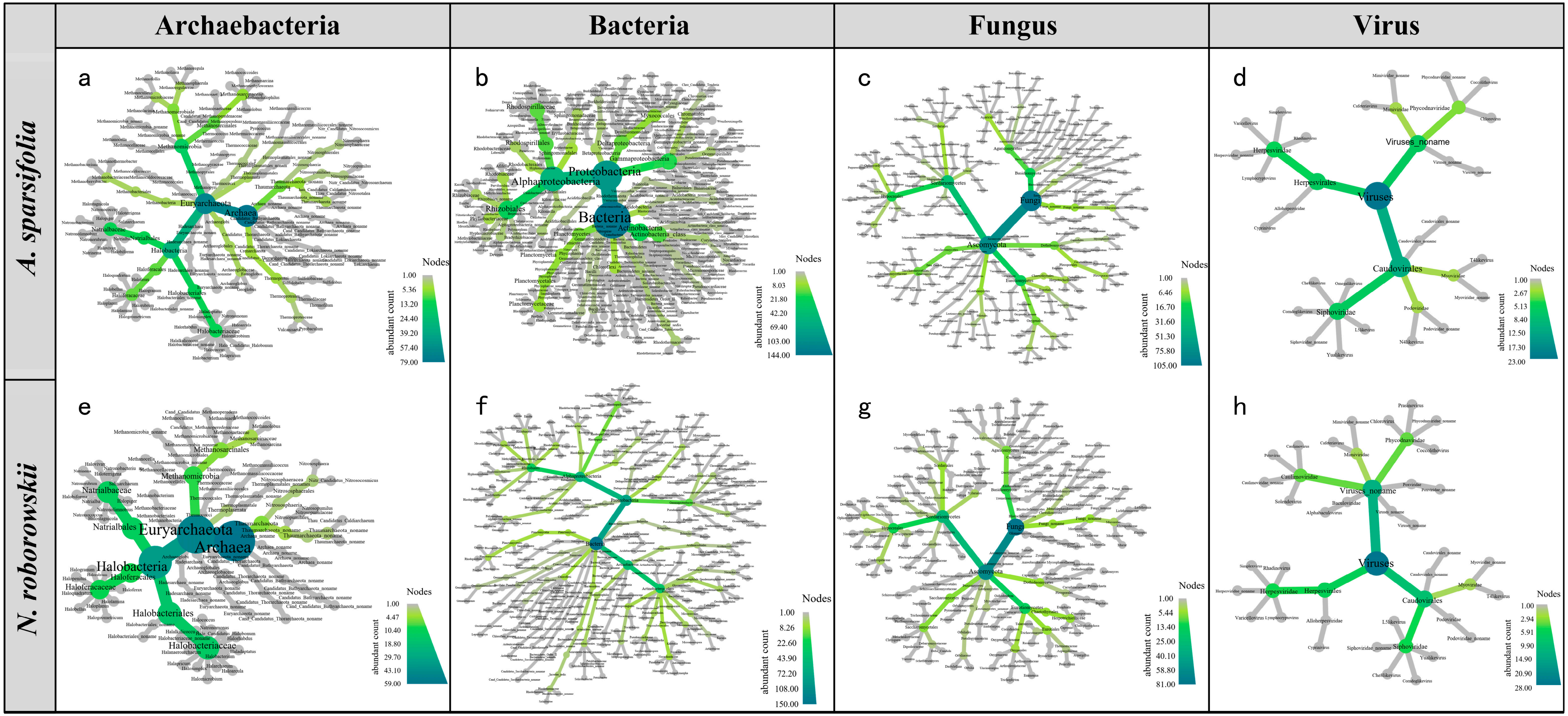

Figure S3. Phylogenetic trees of rhizosphere microbial abundance at genus level: (a-d) *A. sparsifolia* (a) archaea, (b) bacteria, (c) fungi, (d) viruses; (e-h) *N. roborowskii* (e) archaea, (f) bacteria, (g) fungi, (h) viruses.

Table S1. Measurement methods for soil and plant physicochemical properties.

Parameters		Indicators	Experimental method
Soil	C	Soil organic carbon	Potassium dichromate dilution method
	N	Soil total nitrogen	Nesslerization
		Soil nitrate nitrogen	Phenol disulfonic acid method
		Soil ammonium nitrogen	Extraction - indophenol blue colorimetric method
	P	Soil total phosphorus	Molybdenum antimony resistance colorimetry
		Soil available phosphorus	Anti-colorimetric method for extraction of molybdenum and antimony
Plant	C	Plant organic carbon	Potassium dichromate dilution method
	N	Plant total nitrogen	Nesslerization
	P	Plant total phosphorus	Molybdenum antimony resistance colorimetry

Table S2. Eigenvalues and cumulative interpretation rates from Multiple Factor Analysis.

**Multiple Factor Analysis**			
Principal component	Eigenvalue	% of var.	Cumulative % of var.
Dim.1	2.751	31.178	31.178
Dim.2	1.539	17.444	48.622
Dim.3	1.153	13.069	61.69
Dim.4	0.947	10.738	72.428
Dim.5	0.614	6.956	79.385
Dim.6	0.428	4.848	84.233
Dim.7	0.371	4.208	88.441
Dim.8	0.313	3.548	91.989
Dim.9	0.22	2.495	94.484
Dim.10	0.149	1.686	96.17
Dim.11	0.123	1.398	97.568
Dim.12	0.089	1.012	98.58
Dim.13	0.061	0.693	99.273
Dim.14	0.03	0.339	99.612
Dim.15	0.021	0.242	99.854
Dim.16	0.01	0.118	99.973
Dim.17	0.002	0.027	100
